# The amount of DNA combined with TP53 mutations in liquid biopsy is associated with clinical outcome of renal cancer patients treated with immunotherapy and VEGFR-TKIs

**DOI:** 10.1186/s12967-022-03557-7

**Published:** 2022-08-16

**Authors:** Marzia Del Re, Stefania Crucitta, Federico Paolieri, Federico Cucchiara, Elena Verzoni, Francesco Bloise, Raffaele Ciampi, Chiara Mercinelli, Annalisa Capuano, Liberata Sportiello, Antonia Martinetti, Giuseppe Procopio, Luca Galli, Camillo Porta, Sergio Bracarda, Romano Danesi

**Affiliations:** 1 Unit of Clinical Pharmacology and Pharmacogenetics, Department of Clinical and Experimental Medicine, University of Pisa, Pisa, Italy; 2 Unit of Medical Oncology, Department of Translational Research and New Technologies in Medicine and Surgery, University of Pisa, Pisa, Italy; 3 Department of Medical Oncology, Fondazione IRCCS Istituto Nazionale dei Tumori, Milan, Italy; 4 Endocrine Unit, Department of Clinical and Experimental Medicine, University of Pisa, Pisa, Italy; 5 Department of Experimental Medicine, University of Campania “Luigi Vanvitelli”, Naples, Italy; 6 Division of Oncology, Department of Biomedical Sciences and Human Oncology, University of Bari, Bari, Italy; 7 Unit of Medical and Translational Oncology, Department of Oncology, Civil Hospital of Terni, Terni, Italy

**Keywords:** Metastatic clear cell renal cell carcinoma, Biomarkers, Liquid biopsy, Circulating free DNA, TP53, Immunotherapy, Angiogenesis, Tyrosine kinase inhibitors, Predictive biomarkers, Next generation sequencing

## Abstract

**Background:**

Despite the increasing number of treatment options, reliable prognostic/predictive biomarkers are still missing for patients affected by metastatic clear cell renal cell carcinoma (mccRCC).

**Methods:**

Patients with mccRCC undergoing standard first line treatment were enrolled. Blood (12 ml) was drawn at treatment baseline and circulating free DNA (cfDNA) was extracted from plasma. Next-generation sequencing (NGS) was performed on cfDNA using the Oncomine Pan-Cancer Cell-Free Assay and clinical outcomes were correlated with liquid biopsy findings.

**Results:**

A total of 48 patients were enrolled, 12 received immunotherapy and 36 received a vascular endothelial growth factor receptor (VEGFR) tyrosine kinase inhibitor (TKI). A cfDNA cut-off of 0.883 ng/μl stratified patients based on progression-free survival (PFS) and overall survival (OS) (p = 0.001 and p = 0.008, respectively). cfDNA amount was also correlated with best response (p = 0.006). Additional cfDNA cut-points divided patients into short, intermediate and long responders, with PFS of 4.87 vs 9.13 vs 23.1 months, respectively (p < 0.001). PFS resulted to be significantly shorter in carriers of mutant TP53 compared to not carriers (p = 0.04). Patients with high cfDNA levels and mutant TP53 have the worst PFS, while patients with low cfDNA amounts and no mutations in TP53 displayed the longest PFS (p = 0.004).

**Conclusions:**

The present study demonstrates that cfDNA and TP53 are potential predictive biomarkers of response in mccRCC to be further explored in larger and/or prospective studies.

**Supplementary Information:**

The online version contains supplementary material available at 10.1186/s12967-022-03557-7.

## Background

Despite the increasing armamentarium of treatment options for metastatic clear cell renal cell carcinoma (mccRCC), its management still represents a medical challenge owing to the lack of predictive biomarkers for selection of treatments [1, 2]. Compelling evidence demonstrated that mccRCC is an angiogenesis-dependent disease where overproduction of VEGF is the results of molecular abnormalities, including mutations of the von Hippel Lindau (VHL) gene [3]. VHL loss-of-function is the most common genetic finding and is associated with the accumulation of hypoxia-inducible factor (HIF), which results in the excessive production of proangiogenic factors such as vascular endothelial (VEGF), fibroblast (FGF), and platelet-derived (PDGF) growth factors. The availability of VEGFR-tyrosine kinase inhibitors (TKI) alongside with mTOR inhibitors (mTORI) has increased the medical options for the management of mccRCC patients. On the other side, the introduction of anti-PD-(L)1 and anti-CTLA-4 antibodies, and the combination of immunotherapy and VEGFR-TKI positively revolutionized the clinical scenario, posing a new challenge for biomarker discovery. At the moment, apart from guidelines established on the basis of clinical trials, there is a substantial lack of predictive/prognostic biomarkers to support the clinician in her/his therapeutic decision [4]. Candidate genes for a pharmacogenomic stratification of mccRCC include the oncosuppressors TSC1/2 and PTEN, the tumor-drivers PI3K, AKT and mTOR as well as the growth factors released by tumor cells and acting on both the stroma and cancer cells in a paracrine/autocrine fashion, including FGF, PDGF and VEGF. mTOR signaling pathway plays a crucial role in cell growth, survival, proliferation and angiogenesis. PI3K is activated by receptor tyrosine kinases such as VEGFR and insulin-like growth factor receptor (IGFR); the activation of them results in a kinase cascade through AKT and mTOR. This pathway is negatively regulated by the tumor suppressor gene PTEN through the dephosphorylation of phosphatidylinositol (3, 4, 5) trisphosphate (PIP3). Genetic alterations of mTOR pathway-related genes, including PI3K, AKT and PTEN, facilitate tumorigenesis and are common in human cancers. However, recent published results failed to identify and validate predictive biomarkers of response in mccRCC patients [5]. The identification of biomarkers is often based on data obtained from tissue samples including biopsies, an invasive approach compared to liquid biopsy. The analysis of circulating free DNA (cfDNA) offers an alternative, non-invasive tool to interrogate the genetic profile of tumors [6]. Previous studies already suggested cfDNA as an accurate prognostic and predictive biomarker for metastatic cancers [6]; however it has not been validated in mccRCC [7]. In the present study, we aimed to enhance our understanding of the genomic context of mccRCC and the predictive/prognostic value of cfDNA in this disease.

## Patients and methods

The present pharmacogenetic study enrolled patients affected by mccRCC receiving first line treatment with VEGFR-TKIs or a combination of immunotherapy (ipilimumab plus nivolumab) as per approved label. The study was authorized by the Ethics Committee of Pisa University Hospital and conducted in accordance with the principles of Helsinki Declaration. All patients gave their signed informed consent before blood collection and data analysis. Blood samples were drawn from each patient for the analysis of cfDNA before treatment started (baseline). Complete (CR) and partial (PR) responses, disease stabilisation (SD) and progression (PD) were defined following the RECIST criteria (v.1.1).

## Blood sampling and cfDNA isolation

Blood specimens of 12 ml were collected at treatment baseline in EDTA tubes and centrifuged for 10 min at 1900 g within 2 h from sampling. cfDNA isolation was performed on 4 ml plasma from each patient using the MagMAX™ Cell-free Total Nucleic Acid Isolation Kit (ThermoFisher Scientific, Carlsbad, CA) according to the manufacturer’s instructions. Yields of isolated cfDNA were assessed using a Qubit fluorometer and Qubit dsDNA High Sensitivity Assay Kit (Invitrogen, Carlsbad, CA, USA).

## Next-generation sequencing

The Oncomine Pan-Cancer Cell-Free Assay (Thermo Fisher Scientific, Carlsbad, CA) was used to generate libraries from the cfDNA, following the manufacturer’s instructions. The panel covered 52 genes, including hot spots (single nucleotide variants [SNVs] and short indels), copy number variations (CNVs) and gene fusions. Unique index tags were added to each fragment during library preparation. The concentration of the libraries was assessed by quantitative PCR (qPCR) with the Ion Library TaqMan Quantification Kit (Thermo Fisher Scientific, Carlsbad, CA). The quantified stock libraries were then diluted to 100 pM for downstream template preparation. The Ion Chef System and the Ion 540 Kit-Chef were used for template preparation, followed by sequencing on the Ion S5 system using Ion 540 chips. A four-plex library pool was applied to the Ion 540 chip. Raw data were processed automatically on the Torrent Server™ and aligned to the reference hg19 genome. The sequencing data were then analysed by the Ion Reporter™ Analysis Server v. 5.16.0.3 (Thermo Fisher Scientific, Carlsbad, CA) for variant calling and annotation using the workflow Oncomine TagSeq Pan-Cancer Liquid Biopsy w2.4 (Thermo Fisher Scientific, Carlsbad, CA).

## Statistical analysis

Categorical variables were described by absolute and relative frequencies while quantitative factors by median and range. To compare quantitative with categorical variables, the Mann–Whitney test-two tailed was performed. Patients were grouped in two categories based on their best clinical response: patients who experienced CR/PR/SD were defined as “responders”, while patients with PD were defined as “non-responders”. The median cut-off value for analysis of cfDNA was calculated by the Receiver Operating Characteristic (ROC) curve and by the Youden’s Index analysis. PFS and OS were defined as the time from treatment start to progression disease or death, respectively. The Kaplan–Meier method was used to create survival curves and log-rank test was used to evaluate the differences between curves. The Cox hazard regression method was used to identify independent risk factors for OS and PFS. Spearman correlation coefficient was used to assess the correlation between ctDNA amount and the number of metastatic sites or number of mutations detected. Logistic regression model test (Cox-Snell’s R^2^) and ROC curve analysis was computed to estimate diagnostic performance of such signatures, alone and combined with the other biomarkers analysed. χ^2^-test was used to determine the association between cfDNA and PFS and differences were considered significant at p < 0.05. All statistical analyses were performed with MedCalc Statistical Software version 14.8.1 (MedCalc Software, Ostend, Belgium) and the free and open statistical software program JAMOVI^®^ (Version 1.1.9; downloaded from https://www.jamovi.org).

## Results

### Clinical characteristics

Forty-eight patients were enrolled in the present study from June 2018 to December 2019, and clinical characteristics are reported in Table 1. Patients were assigned to treatment as per clinical practice as follows: 12 patients were given ipilimumab-nivolumab combination, and groups of 12 patients each were treated with sunitinib, pazopanib, and cabozantinib. Median PFS was 13.7 months across all treatment arms and median OS was not reached. The comparison between the PFS and OS of single treatments did not reveal any difference (p = 0.9 and p = 0.7, respectively).

**Table 1:** Clinical characteristics of patients and distribution across treatment groups

	Total of patients (n = 48)	Nivolumab + Ipilimumab (n = 12)	Pazopanib (n = 12)	Sunitinib (n = 12)	Cabozantinib (n = 12)	p-value
Age at diagnosis, median (range)	70.5 (46–83)	64 (51–83)	76.5 (50–83)	64 (48–77)	73 (46–76)	–
Gender						0.03
Male	36 (75%)	8 (66.7%)	6 (50%)	12 (100%)	10 (83.3%)	
Female	12 (25%)	4 (33.3%)	6 (50%)	–	2 (16.7%)	
ECOG						0.08
0	28 (58.3%)	6 (50%)	7 (58.3%)	3 (25%)	2 (16.7%)	
1	18 (37.5%)	6 (50%)	5 (41.7%)	9 (75%)	8 (66.7%)	
2	2 (4.17%)	–	–	–	2 (16.7%)	
Stage at diagnosis						0.36
I	5 (10.4%)	3 (25%)	–	4 (33.3%)	–	
II	6 (12.5%)	2 (16.7%)	2 (16.7%)	1 (8.3%)	1 (8.3%)	
III	7 (14.6%)	2 (16.7%)	2 (16.7%)	2 (16.7%)	2 (16.7%)	
IV	30 (62.5%)	5 (41.7%)	8 (66.7%)	5 (41.7%)	9 (75%)	
Nephrectomy						0.025
Yes	29 (60.4%)	6 (50%)	11 (91.7%)	8 (66.7%)	4 (33.3%)	
No	19 (39.6%)	6 (50%)	1 (8.3%)	4 (33.3%)	8 (66.7%)	
Radiotherapy						0.36
Yes	7 (14.6%)	2 (16.7%)	3 (25%)	2 (16.7%)	–	
No	41 (85.4%)	10 (83.3%)	9 (75%)	10 (83.3%)	12 (100%)	
Metastatic sites						0.99
Lung	28 (58.3%)	8 (66.7%)	8 (66.7%)	4 (33.3%)	8 (66.7%)	
Lymph Nodes	23 (47.9%)	6 (50%)	6 (50%)	5 (41.7%)	6 (50%)	
Bones	18 (37.5%)	5 (41.7%)	3 (25%)	5 (41.7%)	4 (33.3%)	
Peritoneus	8 (16.6%)	2 (16.7%)	1 (8.3%)	4 (33.3%)	1 (8.3%)	
Liver	5 (10.4%)	2 (16.7%)	1 (8.3%)	–	2 (16.7%)	
CNS	4 (8.3%)	1 (8.3%)	1 (8.3%)	1 (8.3%)	1 (8.3%)	
Adrenal	6 (12.5%)	2 (16.7%)	–	3 (25%)	1 (8.3%)	
Other	19 (39.5%)	3 (25%)	4 (33.3%)	7 (58.3%)	5 (41.7%)	

### cfDNA as biomarker

**Fig. 1:**
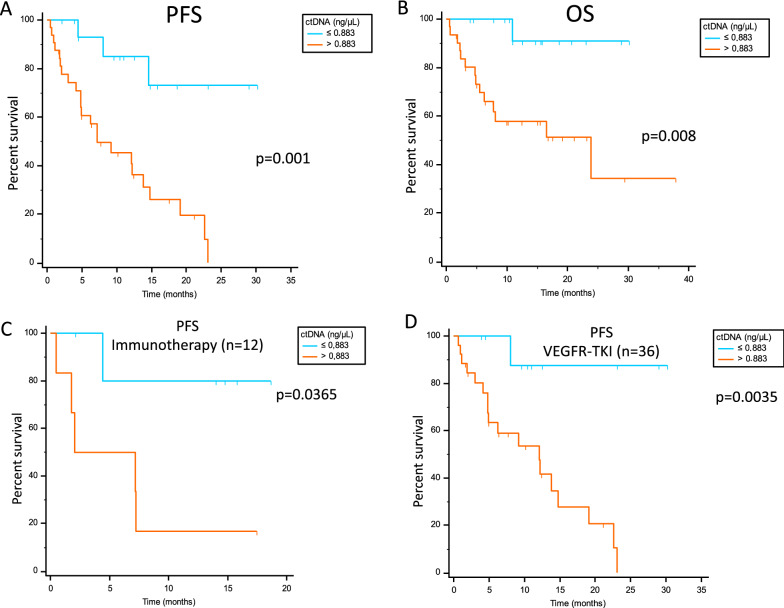
PFS and OS according to cfDNA best cut-off value in the overall population (**A** and **B**), and PFS in immunotherapy (**C**) and in VEGFR-TKI (**D**) treated patients

The ROC curve identified a cfDNA cut off of 0.883 ng/μl as the best value to stratify patients as responders and not responders. Considering the overall population, median PFS was 7.23 months vs not reached in patients with cfDNA > 0.883 ng/μl vs ≤ 0.883 ng/μl (p = 0.001; Fig. 1A). The same finding was obtained for median OS, with 23.9 months vs not reached in patients with > 0.883 ng/μl vs ≤ 0.883 ng/μl (p = 0.008; Fig. 1B). The significance was also maintained in immunotherapy and in VEGFR-TKI-treated patients analyzed separately (Fig. 1C, D).

**Fig. 2:**
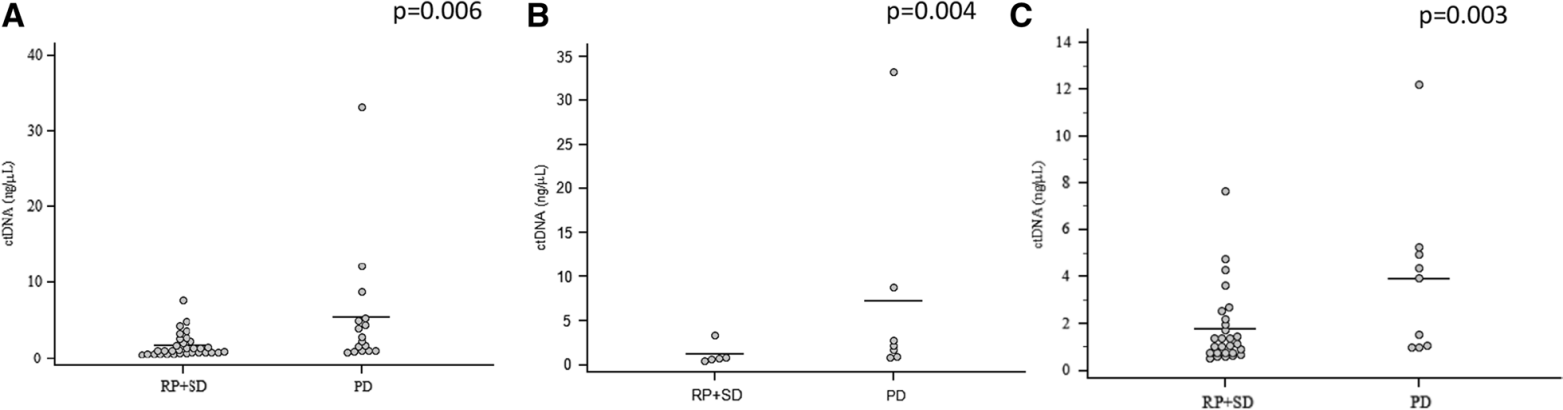
cfDNA levels and its association with best response of patients in the overall population (**A**) and in immunotherapy (**B**) and VEGFR-TKIs (**C**) treated cohorts

**Fig. 3:**
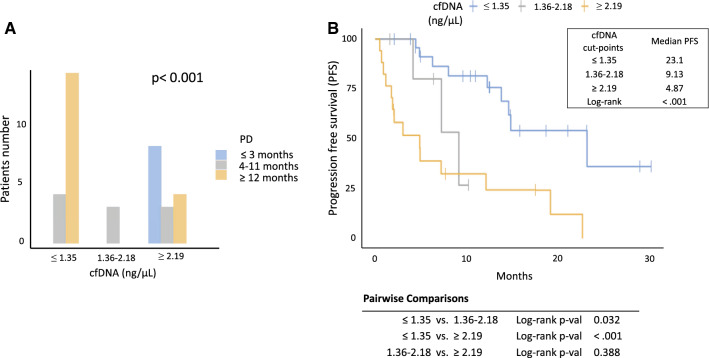
Stratification of patients into short (blue column), intermediate (grey column) and long (orange column) responders, according to the cfDNA cut-points (**A**). Median PFS of short (blue line), intermediate (grey line) and long (orange line) responders, according to the cfDNA cut-points (**B**)

A statistically significant correlation was also found between cfDNA amount and the number of metastatic sites; in particular, higher cfDNA levels were found in patients with more than 4 metastatic sites (p = 0.0073; Additional file 1: Fig. S1). No significant correlation was found between cfDNA levels and the presence or not of the primitive tumor (p = 0.08). Multivariate analysis confirmed the value of cfDNA as unique independent predictive biomarker for PFS, while for OS, cfDNA and ECOG resulted both independent prognostic factors (Additional file 2: Table S1A and Additional file 3: Table S1B). cfDNA amount was also found to be associated with best response in the overall population (p = 0.006, Fig. 2A) as well as with immunotherapy (p = 0.004, Fig. 2B) and VEGFR-TKIs (p = 0.003, Fig. 2C). In detail, responders had lower amount of cfDNA compared to non-responders. Logistic regression showed that cfDNA abundance was also associated with early disease progression, that is, within 3 months from the beginning of treatment (p < 0.001). Therefore, optimal cut-points to identify early progressors and long responders (PFS longer than 12 months) were investigated by using the Youden's index. For early progressors, a cfDNA cut point of ≥ 2.19 ng/μl was identified with 100% sensitivity, 75% specificity, a positive predictive value (PPV) of 53% and a negative predictive value (NPV) of 100% (Youden’s = 0.75). For long responders, a cut-off of ≤ 1.35 ng/μl with a 78% sensitivity, 78% specificity, a PPV of 78% and a NPV of 78%, was defined (Youden’s = 0.556). Twelve patients not reaching 12 months follow-up were removed from the analysis, and patients were divided into 3 groups (short, intermediate and long responders) according to the cfDNA cut-points, with a median PFS of 4.87 vs 9.13 vs 23.1 months, respectively (p < 0.001; Fig. 3A, B).

### Molecular analysis

**Fig. 4:**
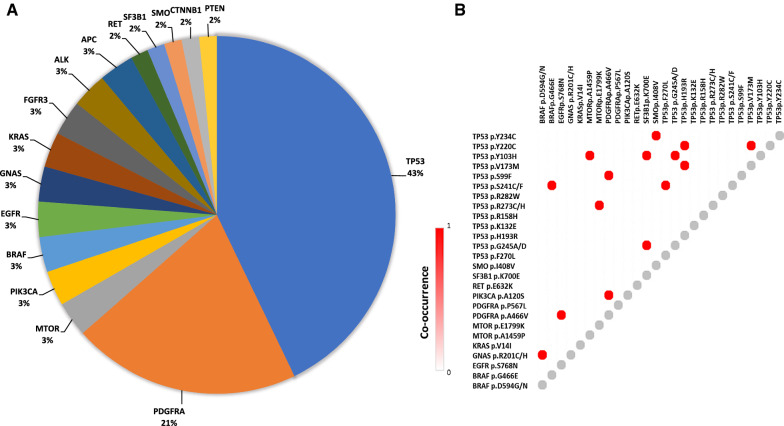
Frequency of mutations (**A**) and their co-occurrency (**B**)

**Fig. 5:**
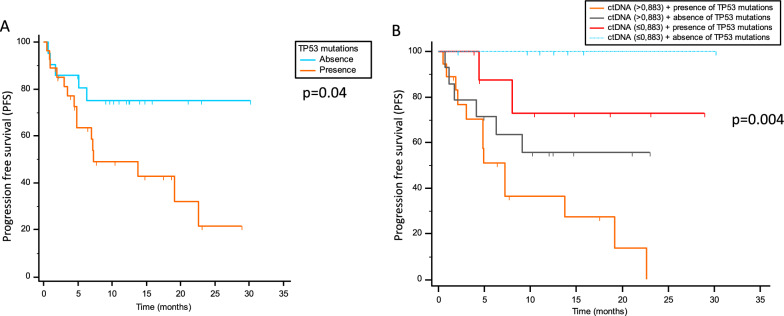
PFS of patients evaluated as carriers vs not carriers of one or more TP53 mutation (**A**). PFS of patients stratified as carriers or not of TP53 mutations and their cfDNA amount (**B**)

The most frequently mutated genes in cfDNA were TP53 (43%) and PDGFRA (21%), followed by mTOR, PI3K, BRAF, EGFR, RET, GNAS, SF3B1, SMO, KRAS, FGFR3, FBXW7, ALK, CTNNB1, PTEN, and APC. The NGS panel used did not include VHL somatic mutations. Mutation distribution and co-occurrency are reported in Fig. 4A and B, respectively. No sensitizing mutations (i.e. BRAF, RET) have been identified that could suggest the use of target-specific drugs. Since TP53 was the most commonly mutated gene found in the overall population, PFS was evaluated in carriers vs not carriers of one or more TP53 mutations. PFS resulted to be shorter in carriers compared to not carriers (p = 0.04, 7.2 months vs not reached, respectively; Fig. 5A). Finally, PFS was evaluated by stratifying patients taking into account both TP53 mutational status and cfDNA levels, and was found that subjects with high cfDNA concentrations and mutated TP53 had the worst PFS, while patients with low cfDNA and no mutations in TP53 had the longer PFS (p = 0.004; Fig. 5B).

## Discussion

In this hypothesis-generating exploratory analysis, cfDNA amount and TP53 status were found to be associated with first line treatment outcome in mccRCC. Although the available therapeutic options of mccRCC are expanding [8], a personalized treatment is still limited due to a lack of validated predictive/prognostic biomarkers. Recent evidence demonstrated the feasibility of cfDNA analysis and its high concordance with tissue genomic analysis in mccRCC patients [9]. cfDNA assessment has numerous advantages over tissue biopsies, including minimal invasiveness, the ability to capture intratumoral heterogeneity, and the feasibility of repeated assessments of genomic profile to track tumor dynamics [10–12]. Published studies already demonstrated the role of cfDNA to perform initial diagnosis or detecting disease recurrence [13, 14]. Moreover, early variations in cfDNA levels are known to be associated with response to treatment [15, 16]. The present study was aimed at evaluating the association of cfDNA and genomic alterations with response in 48 mRCC patients undergoing treatment with VEGFR-TKIs or immunotherapy. The study involved 12 patients treated with nivolumab plus ipilimumab, and 36 patients treated with TKIs, including sunitinib, pazopanib and cabozantinib. The PFS univariate models showed no significant differences concerning treatment types while ctDNA amount was significantly associated with PFS, regardless of the therapeutic management. This result is consistent with the one from Yamoto et al. [16], which found that cfDNA levels were significantly associated with PFS and cancer-specific survival, suggesting its potential role as a biomarker in ccRCC patients with or without metastases. Moreover, other published data suggested an association between cfDNA detection and high radiographic tumor burden [17]. A correlation was also found between the number of metastatic sites and cfDNA levels, thus suggesting a potential role for cfDNA in monitoring response to treatment with detectable changes occurring before radiographic evidence of disease progression [17].

Importantly, the predictive value of cfDNA as an independent predictive biomarker was maintained at the multivariate analysis. Cut-off points enabled us to identify three groups of patients at high, intermediate or low risk to progress to treatment, potentially allowing clinicians to avoid expensive treatments in patients who will not respond to therapy. The clinical utility of cfDNA for the management of patients affected by different tumor types and treated with immunotherapy, targeted therapies or chemotherapy has been demonstrated in several studies [18], including melanoma [19], colorectal cancer [20], lung [21] and breast cancer [22]. However, despite increasing evidence in favor of the predictive and prognostic role of cfDNA, there is still a lack of consensus for its assessment in clinical practice. Seventeen genes were found mutated alone or concurrently in the present population, including TP53, mTOR, PI3K, BRAF, EGFR, RET, GNAS, SF3B1, SMO, KRAS, PDGFRA, FGFR3, FBXW7, ALK, CTNNB1, PTEN, and APC. Being a pan-cancer assay, VHL somatic mutations were not included in the NGS panel used in this study. However, despite its clear role in ccRCC, VHL mutations are not considered a biomarker, neither tested during the diagnostic work-up [23, 24]. Unfortunately, no sensitizing mutations predictive of response to targeted treatment (i.e. BRAF p.V600E; MET amplification) were detected in this study. TP53 was the most common mutant gene in the overall population, and was significantly associated with PFS, alone or in combination with cfDNA. It is worth to note that ccRCC harboring TP53 alterations showed deregulation of cell cycle, FAS/pentose phosphate pathway and stromal gene expression [25]. In addition to this, it was reported that TP53 downregulates HIF-1α in RCC [26], and tumors with loss-of-function mutations of TP53 show enhanced vascular growth. Therefore, the findings of the present study demonstrating an association of TP53 mutations with shorter PFS are justified by the relevant biologic role of TP53 loss-of-function on cell cycle and angiogenesis, including immunosuppression induced by overproduction of VEGF [27].

The present study adds new evidence in favor of the potential usefulness of cfDNA in monitoring mccRCC response to treatment. It has been demonstrated that cfDNA and tissue-based genomic profiling are complementary to identify actionable alterations in mRCC [28]. Furthermore, cfDNA can detect changes in genomic profiles over time and may potentially help in guiding treatment. Analysis of large cohorts of patients affectd by ccRCC demonstrated significant changes in cfDNA during the clinical course of the disease, which may have therapeutic implications [29]. Since collection of cfDNA is feasible in patients with metastatic tumors, this test can be easily translated also into the clinical practice of mccRCC, as it has been already done in different tumors, such as lung cancer [30].

The concordance of cfDNA with radiological imaging is also already known in different tumor types, including lung, breast, and bladder cancer [31, 32]. In particular, it is known that cfDNA variations are able to anticipate tumor radiological and clinical progression by weeks. However, how to practically translate this information in clinical practice awaits prospective validation. Since new drug combinations may improve survival in patients with mccRCC, cfDNA levels may integrate the stratification-risk algorithm of patients, allowing for an easier choice of treatments, based on the hypothesis that high cfDNA amount may suggest a more biologically aggressive disease that may benefit from a drug combination or a more intensive therapeutic approach.

## Conclusions

The major limitation of the present retrospective study is the small and heterogeneous cohort of patients; nonetheless, it provides the first evidence that liquid biopsy may be used to identify biomarkers associated with response or resistance to treatments in mccRCC. These preliminary findings, if validated in larger prospective cohort, can help the clinician improve the selection of patient candidates for treatment and/or monitor their clinical outcome.

## Supplementary Information

**Additional file 1: Figure S1.** Correlation between cfDNA amount and the number of metastatic sites.

**Additional file 2: Table S1A.** Univariate and multivariate analysis for PFS.

**Additional file 3: Table S1B.** Univariate and multivariate analysis for OS.

## Data Availability

The datasets used and/or analysed during the current study are available from the corresponding author upon reasonable request.

## Abbreviations

cfDNA: Circulating free DNA
CNV: Copy number variation
CR: Complete response
FGF: Fibroblast growth factor
HIF: Hypoxia-inducible factor
IGFR: Insulin-like growth factor receptor
mccRCC: Metastatic clear cell renal cell carcinoma
mTORI: MTOR inhibitor
NGS: Next-generation sequencing
NPV: Negative predictive value
OS: Overall survival
PD: Disease progression
PDGFRA: Platelet-derived growth factor receptor alpha
PFS: Progression-free survival
PIP3: Phosphatidylinositol (3, 4, 5)-trisphosphate
PPV: Positive predictive value
PR: Partial response
qPCR: Quantitative PCR
ROC: Receiver operating characteristic
SD: Stable disease
SNV: Single nucleotide variant
TKI: Tyrosine kinase inhibitor
VEGFR: Vascular endothelial growth factor receptor
VHL: Von Hippel Lindau
