# Supplementary figures and images for: The amount of DNA combined with TP53 mutations in liquid biopsy is associated with clinical outcome of renal cancer patients treated with immunotherapy and VEGFR-TKIs
Source: J Transl Med. 2022 Aug 16;20:371. doi: 10.1186/s12967-022-03557-7 (PMC9382729; doi:10.1186/s12967-022-03557-7)

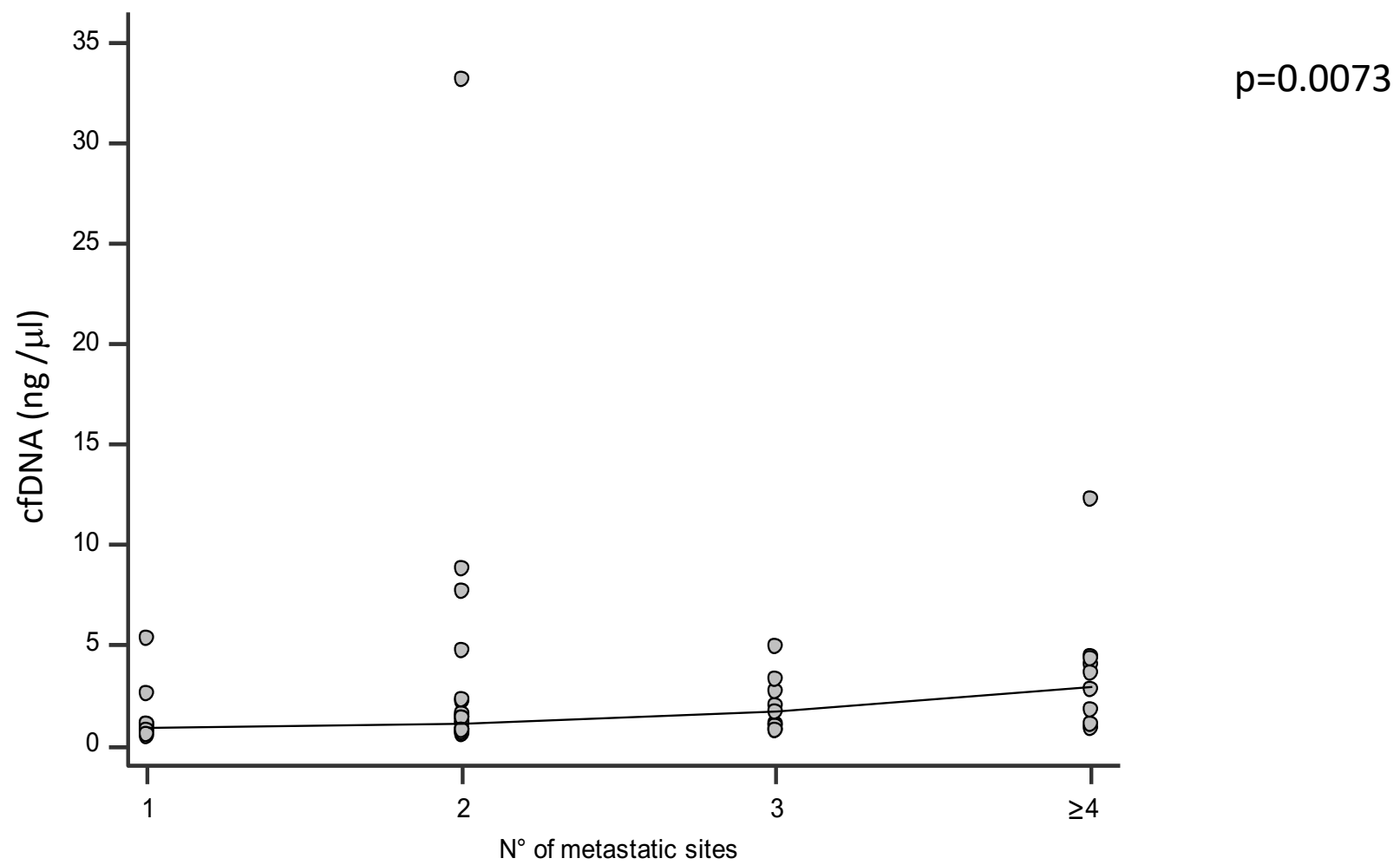

Figure S1

Supplement: Supplementary file 1 — Additional file 1: Figure S1. Correlation between cfDNA amount and the number of metastatic sites. [file 12967_2022_3557_MOESM1_ESM.pdf]
