# Supplementary material for: The amount of DNA combined with TP53 mutations in liquid biopsy is associated with clinical outcome of renal cancer patients treated with immunotherapy and VEGFR-TKIs
Source: J Transl Med. 2022 Aug 16;20:371. doi: 10.1186/s12967-022-03557-7 (PMC9382729; doi:10.1186/s12967-022-03557-7)
Supplement: Supplementary file 2 — Additional file 2: Table S1A. Univariate and multivariate analysis for PFS. [file 12967_2022_3557_MOESM2_ESM.docx]

**Additional file 2: Table S1A.** Univariate and multivariate analysis for PFS

|  | Univariate | | Multivariate | |
| --- | --- | --- | --- | --- |
| Variables | HR (95% CI) | p-value | HR (95% CI) | p-value |
| Gender | 1.92 (0.80 – 4.65) | 0.15 | - | - |
| Age | 1.02 (0.98 – 1.05) | 0.36 | - | - |
| ECOG | 9.97 (2.07 – 48.05) | **0.004** | 4.92 (0.97 – 24.91) | 0.06 |
| Stage at diagnosis | 1.49 (0.95 – 2.32) | 0.08 | - | - |
| Nephrectomy | 0.40 (0.18 – 0.91) | **0.03** | 0.53 (0.23 – 1.26) | 0.15 |
| Radiotherapy | 0.94 (0.32 – 2.74) | 0.92 | - | - |
| Number of metastatic sites | 1.31 (0.91 – 1.90) | 0.15 | - | - |
| ctDNA  (≤0.883, >0.883) | 6.07 (1.82 – 20.27) | **0.003** | 5.38 (1.59 – 18.19) | **0.007** |
