# Supplementary material for: The amount of DNA combined with TP53 mutations in liquid biopsy is associated with clinical outcome of renal cancer patients treated with immunotherapy and VEGFR-TKIs
Source: J Transl Med. 2022 Aug 16;20:371. doi: 10.1186/s12967-022-03557-7 (PMC9382729; doi:10.1186/s12967-022-03557-7)
Supplement: Supplementary file 3 — Additional file 3: Table S1B. Univariate and multivariate analysis for OS. [file 12967_2022_3557_MOESM3_ESM.docx]

**Additional file 3: Table S1B.** Univariate and multivariate analysis for OS

|  | Univariate | | Multivariate | |
| --- | --- | --- | --- | --- |
| Variables | HR (95% CI) | p-value | HR (95% CI) | p-value |
| Gender | 1.80 (0.57 – 5.74) | 0.32 | - | - |
| Age | 1.02 (0.98 – 1.07) | 0.30 | - | - |
| ECOG | 13.40 (2.59 – 69.45) | 0.002 | 6.13 (1.10 – 33.99) | **0.04** |
| Stage at diagnosis | 1.30 (0.77 – 2.18) | 0.33 | - | - |
| Nephrectomy | 0.33 (0.11 – 0.98) | 0.05 | 0.47 (0.15 – 1.49) | 0.20 |
| Radiotherapy | 0.37 (0.05 – 2.84) | 0.35 | - | - |
| Number of metastatic sites | 1.05 (0.64 – 1.72) | 0.85 | - | - |
| ctDNA (≤0.883, >0.883) | 9.28 (1.23 – 70.02) | 0.03 | 7.77 (1.02 – 59.41) | **0.05** |
